# Impact of initial dialysis modality on mortality: a propensity-matched study

**DOI:** 10.1186/s12882-015-0175-5

**Published:** 2015-10-30

**Authors:** Bård Waldum-Grevbo, Torbjørn Leivestad, Anna V Reisæter, Ingrid Os

**Affiliations:** Institute of Clinical Medicine, Faculty of Medicine, University of Oslo, Oslo, Norway; Department of Nephrology, Oslo University Hospital, PB 4956 Nydalen, N-0424, Oslo, Norway; Norwegian Renal Registry, Department of Transplantation Medicine, Oslo University Hospital, Oslo, Norway; Section of Nephrology, Department of Transplantation Medicine, Oslo University Hospital, Oslo, Norway

**Keywords:** Dialysis modality, ESRD, Haemodialysis, Peritoneal dialysis, Prognosis, Survival analysis

## Abstract

**Background:**

Whether the choice of dialysis modality in patients with end stage renal disease may impact mortality is undecided. No randomized controlled trial has properly addressed this issue. Propensity-matched observational studies could give important insight into the independent effect of peritoneal (PD) opposed to haemodialysis (HD) on all-cause and cardiovascular mortality.

**Methods:**

To correct for case-mix differences between patients treated with PD and HD, propensity-matched analyses were utilized in all patients who initiated dialysis as first renal replacement therapy in Norway in the period 2005–2012. PD patients were matched in a 1:1 fashion with HD patients, creating 692 pairs of patients with comparable baseline variables. As-treated and intention-to treat analyses were undertaken to assess cardiovascular and all-cause mortality. Interaction analyses were used to assess differences in the relationship between initial dialysis modality and mortality, between strata of age, gender and prevalent diabetes mellitus.

**Results:**

In the as-treated analyses, initial dialysis modality did not impact 2-year (PD vs. HD: HR 0.87, 95 % CI 0.67–1.12) or 5-year all-cause mortality (HR 0.95, 95 % CI 0.77–1.17). In patients younger than 65 years, PD was superior compared to HD with regard to both 2-year (HR 0.39, 95 % CI 0.19–0.81), and 5-year all-cause mortality (HR 0.49, 95 % CI 0.27–0.89). Cardiovascular mortality was also lower in the younger patients treated with PD (5-year HR 0.38, 95 % CI 0.15–0.96). PD was not associated with impaired prognosis in any of the prespecified subgroups compared to HD. The results were similar in the as-treated and intention-to-treat analyses.

**Conclusion:**

Survival in PD was not inferior to HD in any subgroup of patients even after five years of follow-up. In patients below 65 years, PD yielded superior survival rates compared to HD. Increased use of PD as initial dialysis modality in ESRD patients could be encouraged.

## Background

An aging population and increasing incidence of atherosclerotic disease, hypertension and diabetes have caused a substantial rise in patients in need of renal replacement therapy (RRT) in Norway similar to what has been observed in other countries [1, 2]. Peritoneal dialysis (PD) and haemodialysis (HD) are considered equivalent treatment options in end stage renal disease (ESRD). Preserved residual kidney function, reduced infection risk and improved patient contentment, as well as reduced cost and manpower use, are arguments for increased use of PD [3]. Despite that, the proportion of patients treated with PD has remained stable in Norway during the last two decades [1], but has decreased in other countries [4].

Dialysis patients experience a high risk of all-cause and cardiovascular mortality [5, 6]. Whether dialysis modality independently affects mortality is under debate. No randomized controlled trial has been performed to assess the independent effect of HD and PD on mortality, and this is unlikely to ever occur. Observational data suggest that there is no difference in survival between the two modalities [7–11]. Notwithstanding, a survival advantage for PD the first years after dialysis initiation has been reported in young patients without comorbidity, e.g. diabetes [12–15], while HD may be associated with improved survival beyond 1–2 years in dialysis [16] . As the need for RRT is increasing, particularly in developing countries [2], and PD may provide RRT at lower cost and higher patient contentment, there is a need to further clarify whether initial dialysis modality may impact survival.

Observational studies that compare outcome differences between the two dialysis modalities may be limited by confounding by indication. Patient treatment preferences, center experience, cause of ESRD, time of referral to nephrologist, and comorbidity may all affect the choice of initial dialysis modality. Thus, proper adjustment for the case-mix differences between HD and PD patients must be undertaken to solve the problem of this bias. Propensity score matched analyses mimic some of the characteristics of a randomized controlled trial and may better correct for case-mix differences than traditional regression analyses [17].

The aims of the current study were to utilize propensity matched analyses to assess all-cause and cardiovascular mortality in patients treated with PD compared to HD as initial RRT. Furthermore, the aim was to investigate if PD or HD were superior with respect to mortality in any strata of age, gender and prevalent diabetes mellitus.

## Methods

### Study population

The Norwegian Renal Registry is a national registry comprising all ESRD patients residing in Norway who receive RRT, both renal transplantation and chronic dialysis. Patients are included in the registry when they first start chronic dialysis treatment or receive a renal transplant. Changes in RRT are recorded at the time of the event, as is cessation of dialysis treatment, emigration and death. Dialysis patients have been included in the national registry from 1980. From 2005, patients starting dialysis as first RRT were registered with more comprehensive data of baseline characteristics including laboratory values and comorbidity. Adult patients (>18 years) who started dialysis as initial RRT in the time period from January 2005 through December 2012 were included in the current study. The study database contained only 0.55 % missing data. No patient was lost to follow-up.

All participants provided written informed consent before inclusion in the registry. Permission for the current study was granted from the Regional Committee of Medical and Health Research.

### Definitions

Primary renal diagnosis was classified according to the ERA-EDTA primary renal disease codes, and ERA-EDTA cause of death (COD) codes were applied [18]. Cardiovascular cause of death was defined as a composite of COD group I–IV including myocardial ischemia and infarction, heart failure, cardiac arrest/sudden death and cerebrovascular accident [18].

Renal function at the time of dialysis initiation was expressed as estimated glomerular filtration rate (eGFR) using MDRD formula.

Previous heart disease was defined as a diagnosis of coronary heart disease and/or heart failure prior to dialysis initiation. Left ventricular hypertrophy (LVH) should have been confirmed by echocardiography. Previous cerebrovascular disease (CVD) included previous cerebrovascular insult or transischemic attack (TIA). Peripheral arterial disease (PAD) was defined as established atherosclerotic vascular disease other than vascular disease in the brain or heart, e.g. aortic aneurysm, renovascular disease and intermittent claudication. Any history of a malignant tumour diagnosis prior to dialysis treatment was registered as previous malignancy.

Norway consists of 19 counties, both urban and rural areas, some scarcely populated. Use of PD has varied substantially between the counties through the years [1], and this is most likely caused by the difference in experience and traditions in the renal units. Therefore, patients were propensity matched also according to county affiliation.

### Statistical analyses

Continuous variables were presented as mean ± SD, or median and range if skewed. Categorical data were presented as percentage. Student *t*-tests were used to compare normally distributed continuous variables between patients in PD and HD. Mann-Whitney U-test was used if data were skewed. Chi-square test was applied to compare categorical data.

A time dependent logistic regression model was built to create propensity scores of being treated with PD compared to HD as the initial dialysis modality. As the population started in PD may have changed in the study period due to a challenged HD capacity in Norway, a individual propensity score of being treated with PD was calculated separately in patients started in dialysis in the period 2005 through 2008 and the period 2009 through 2012. Baseline covariates were entered as independent variables in the model: age, gender, county, primary cause of ESRD, comorbidities (diabetes mellitus, LVH, established heart disease, PAD, CVD and previous malignancy), eGFR, haemoglobin, serum albumin, number of antihypertensive drugs, use of statins, erythropoietin stimulating agents, vitamin D supplementation, candidate of future transplantation and crashlanders (knowledge of the patients less than four months prior to start of dialysis). Less than 1 % of the variables were missing. Patients with missing data were excluded from further analyses as a complete data set was mandatory to produce a propensity score. PD patients were matched 1:1 with HD patients by best match using R extension pack (R version 2.12.1) with IBM SPSS Statistics [19].

Survival analyses were conducted both in the terms of as-treated and intention-to-treat. In the as-treated analyses patients were censored for the earliest of the following: renal transplantation, dialysis modality change, dialysis cessation, emigration or study end by December 2012. In the intention-to-treat analyses patients were censored in the same way except that change in dialysis modality did not induce end of follow-up time.

Kaplan-Meier plots and log-rank statistics were used to analyse differences in survival between the initial dialysis modalities in the propensity matched cohort. Hazard ratios were calculated from univariate Cox regression analyses. The assumption of proportional hazard were checked and found to be adequately met.

Stratified analyses were undertaken with respect to age, gender and diabetes mellitus. Differences between the strata were checked by interaction analyses by entering product terms into the Cox regression models.

Level of significance was set to 0.05. Analyses were performed with IBM SPSS statistical software (version 20.0, IBM SPSS Statistics, New York, U.S.).

## Results

A total of 3555 adult patients received dialysis as first active ESRD treatment in Norway in the period from 2005 to 2012. A propensity score could be calculated in 3089 patients who had complete datasets (age 64.8 ± 15.3 years, 67 % men). HD was the most frequent initial dialysis modality (*n* = 2407, 78 %), while 692 patients (22 %) were started in PD. Median as-treated follow-up time was 12 months (range 0–92). The baseline characteristics of the included patients are presented in Table 1. The unmatched case-mix differences between PD and HD patients were substantial.

**Table 1:** Baseline characteristics of Norwegian patients starting peritoneal- or hemodialysis as initial renal replacement therapy in the period 2005–2012

	All	Unmatched HD	Matched HD	PD	*p*-values	
	(*n* = 3089)	(*n* = 2407)	(*n* = 682)	(*n* = 682)	PD vs. unmatched HD	PD vs. matched HD
Age (years)	64.8 (15.3)	64.9 (15.4)	65.2 (15.0)	64.6 (15.2)	NS	NS
Male gender (%)	67.2	67.3	67.0	66.7	NS	NS
County					<0.001	NS
Renal diagnosis					<0.001	NS
BMI kg/m^2^	25.9 (5.2)	26.1 (5.5)	25.5 (5.1)	25.4 (4.2)	0.001	NS
eGFR ml/min/1.73 m^2^	8.3 (3.2)	8.2 (3.2)	9.0 (3.4)	8.8 (3.1)	<0.001	NS
Hb g/dl	10.6 (1.6)	10.4 (1.6)	11.2 (1.5)	11.3 (1.4)	<0.001	NS
Albumin g/l	34.9 (6.7)	34.0 (6.7)	37.8 (5.5)	38.0 (5.7)	<0.001	NS
Prev comorbidities						
DM (%)	32.1	32.9	30.6	29.3	NS	NS
Heart disease (%)	31.6	33.1	30.5	26.5	0.001	NS
LVH (%)	31.3	32.2	28.9	28.2	0.044	NS
CVD (%)	13.9	14.5	12.0	11.9	NS	NS
PAD (%)	20.2	21.2	18.0	16.6	0.008	NS
Malignancy (%)	10.0	11.3	5.4	5.7	<0.001	NS
Medications						
Number of BP medication (%)					<0.001	NS
0	10.1	11.8	3.2	4.1		
1	12.6	12.8	12.0	12.0		
2	22.5	22.3	22.4	23.3		
3	29.4	28.6	33.3	32.3		
4	17.3	16.5	20.1	19.9		
>4	8.1	8.1	8.9	8.4		
Statin (%)	54.0	52.3	61.1	60.3	0.001	NS
Vitamin D (%)	59.1	55.6	70.4	71.4	<0.001	NS
ESA (%)	59.0	55.9	70.8	70.1	<0.001	NS
Known by nephrologist (%)	74.2	69.4	91.1	91.1	<0.001	NS
RTX candidate (%)	59.8	57.0	68.3	69.5	<0.001	NS
Follow-up time (months)	12 (0–92)		13.0 (0–89)	10.0 (0–73)		<0.001

Continuous data are shown as mean ± SD, median (range) if skewed, and categorical data as percentages.

*BMI*: Body mass index, *County*: Patients were categorized due to situation in 19 counties in Norway, *CVD*: cerebrovascular disease, *Diagnosis*: patients were categorized due primary renal diagnosis defined by ERA-EDTA primary renal disease codes, *DM*: diabetes mellitus, *ESA*: erythropoiesis stimulating agents, *Follow-up time:* As-treated follow-up time prior to censoring or death, *Heart disease*: previous diagnosis of coronary heart disease or heart failure, *Known by nephrologist*: treated by nephrologist > 4 months prior to dialysis initiation, *LVH:* left ventricular hypertrophy, *Malignancy*: previous diagnosis of malignant disease, *PAD*: peripheral artery disease, *RTX candidate*: considered suitable for future renal transplantation by treating nephrologist, *vitamin D*: supplementation with active vitamin D or paricalcitol

Patients with PD as initial modality were propensity matched 1:1 with patients who started with HD. The final analyses included 692 matched pairs of patients. The differences in baseline characteristics between PD and HD patients were balanced in the propensity matching procedure (Table 1). The as-treated follow-up time was lower in the PD group compared to the HD group (median 10 (0–73) vs. 13 (0–89) months, *p* < 0.001). The intention-to-treat follow-up time, i.e. patients were not censored for change of dialysis modality, was equal in the two groups (14 (0–95) months in PD vs. 15 (0–89) months in HD, *p* = 0.359).

**Fig. 1:**
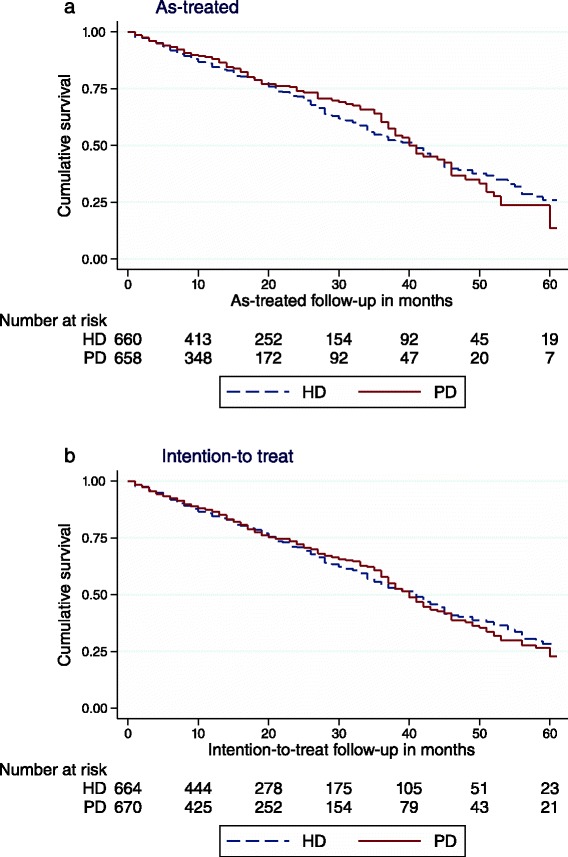
Kaplan-Meier 5-year survival plot comparing peritoneal- and haemodialysis as initial dialysis modality in 692 propensity matched pairs of patients with end stage renal disease in Norway. As-treated, i.e. censored for change of dialysis modality, renal transplantation, dialysis cessation, emigration or end of study (**a**) and intention-to-treat, i.e. censored for renal transplantation, dialysis cessation, emigration or end of study (**b**), analyses

All-cause mortality was not affected by initial dialysis modality in the as-treated analyses, (2-year PD vs. HD HR 0.87, 95 % CI 0.67–1.12 and 5-year HR 0.95, 95 % CI 0.77–1.17, Fig. 1). Neither did dialysis modality impact survival in the intention-to-treat analyses (Table 2).

**Table 2:** Cox regression all–cause mortality analyses comparing PD with HD as initial dialysis modality in 692 pairs of propensity matched patients in Norway

a)						
	2-year all-cause mortality					
	*As–treated*			*Intention–to–treat*		
	Hazard ratio	95 % CI	*p*–value for interaction	Hazard ratio	95 % CI	*p*–value for interaction
All	0.87	0.67–1.12		0.93	0.73–1.18	
Male	0.93	0.67–1.29	NS	1.03	0.77–1.38	NS
Female	0.77	0.50–1.17		0.79	0.53–1.16	
DM-	0.75	0.55–1.02	NS	0.82	0.62–1.09	NS
DM+	1.20	0.76–1.91		1.22	0.80–1.86	
≤65 years	0.39	0.19–0.81	0.011	0.47	0.26–0.85	0.009
>65 years	1.03	0.78–1.36		1.11	0.86–1.44	
b)						
	5-year all-cause mortality					
	*As-treated*			*Intention–to–treat*		
	Hazard ratio	95 % CI	*p*-value for interaction	Hazard ratio	95 % CI	*p*–value for interaction
All	0.95	0.77–1.17		0.99	0.82–1.19	
Male	1.09	0.83–1.44	NS	1.06	0.83–1.34	NS
Female	0.78	0.55–1.09		0.87	0.65–1.19	
DM-	0.91	0.70–1.18	NS	1.02	0.81–1.29	NS
DM+	0.99	0.69–1.42		0.90	0.65–1.25	
≤65 years	0.49	0.27–0.89	0.010	0.58	0.36–0.93	0.009
>65 years	1.06	0.84–1.33		1.13	0.92–1.39	

**Fig. 2:**
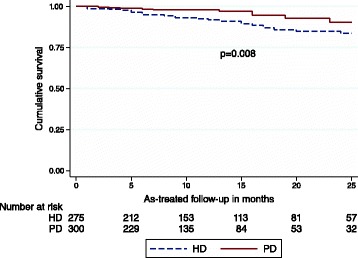
Kaplan-Meier 2-year survival plot comparing peritoneal- and haemodialysis as initial dialysis modality in propensity matched patients younger than 65 years

In the stratified analyses, PD was associated with reduced all-cause mortality in patients aged below 65 years (Fig. 2). The results were similar in both the as-treated and intention-to-treat analyses (Table 2). PD was not associated with impaired survival in any subgroup of patients (Table 2).

Cardiovascular disease was the cause of death in 117 of 238 patients (49.1 %) at 2- year, and in 176 of 365 patients (48.2 %) at 5-year as-treated follow-up. In patients younger than 65 years, 5-year as-treated cardiovascular mortality was lower in PD compared to HD (HR 0.38, 95 % CI 0.15–0.96). This was not observed in patients older than 65 years (p for interaction = 0.023). The intention-to-treat analyses confirmed lower 5-year cardiovascular mortality in the younger (HR 0.28 95 % CI 0.11–0.68).

No interactions between gender or diabetes mellitus and initial dialysis modality were found concerning mortality.

## Discussion

Initial dialysis modality did not impact all-cause or cardiovascular mortality in this propensity matched study. However, the present study suggests that PD was more favourable with respect to survival compared to HD in patients below 65 years. Actually, 2-year all-cause mortality was more than halved in the younger PD patients compared to what was observed in HD patients, and the improved all-cause and cardiovascular survival persisted through five year of follow-up.

PD and HD have been considered to be equally effective as initial dialysis modalities concerning risk of mortality [7–11]. Observational studies have also previously reported favourable outcomes in younger PD patients without comorbidities compared to HD patients, however limited to the first 1–2 years of dialysis treatment [12–15]. The concern about increased mortality in PD compared to HD if treatment is continued beyond 1–2 years is not supported by our study [16]. Furthermore, elderly patients with diabetes mellitus have been claimed to do worse in PD compared to HD [7, 11]. We did not find any interaction between diabetes mellitus and dialysis modality related to outcome. No investigated subgroup had worse outcome in PD compared to HD in the present study, whether using as-treated or intention-to-treat analyses.

There are several reasons why our results may diverge from that of previous studies comparing outcome in PD and HD. Earlier studies are typically based on dialysis registries with patients included in the late 1990s and early 2000s [15, 16, 20]. Although survival for both HD and PD patients has improved during the last decades, survival has improved more in PD patients than in HD patients [21]. Our study included patients in the time period 2005 to 2012. During this period, more physiological dialysis solutions have been utilized for PD patients in Norway. Neutral-pH, low-glucose degradation product PD solutions result in better preservation of residual renal function and greater urine output compared to the traditional solutions [22]. Furthermore, the use of icodextrin and amino acid as alternative to glucose as osmotic agent may improve ultrafiltration capacity and lessen the harmful effects caused by glucose [23]. Thus, one may assume that our findings are partly explained by the evolvement of PD treatment in the last decade, suggesting that patients in the present study received improved quality PD compared to patients in most previous studies.

Propensity matched analyses were used to correct for the case-mix differences between patients treated with HD and PD. Based on predefined measured variables, patients starting PD were matched with HD patients with comparable baseline characteristics decided by propensity score. Propensity matched analyses open the possibility to design and analyse observational data so that it mimics some of the particular characteristics of a randomized controlled trial and could be superior to multivariate Cox regression to correct for confounding factors in observational studies [17]. Randomized controlled trials have been called for to assess the independent effect of dialysis modality on patient survival, but will probably never be performed. Both Cox regression and propensity matched analyses will be unable to properly correct for non-measured confounding variables. However, similar follow-up time in PD and HD patients in the intention-to-treat analyses should indicate that the propensity matched procedure was successful in creating comparable groups in our study.

Compared to similar studies that also used propensity matched analyses, our patients were matched on a larger number of variables and this should ensure improved reliability of our results [14, 24]. A recent American study by Kumar et al. [14] used methods and definitions comparable to ours. In this study, the cumulative HR of death favoured PD for up to three years in the as-treated analyses with no difference therafter [14]. As our study suggested favourable outcomes in PD in the younger patients, the difference in results from our study may be explained by the age difference between the two populations. While the mean age of American PD patients was 57 years, the mean age in our population was 65 years in PD as well as HD patients.

A higher rate of treatment failure is likely the cause of the shorter as-treated follow-up time in PD patients compared to that of HD patients. Technique failure is a major challenge for the “PD-first” policy, and the failure rate has not improved significantly the later years [3, 25]. PD was, however, a safe initial dialysis modality in all subgroups of patients in our study, as mortality rates were not affected by a later need of treatment conversion in our intention-to-treat analyses. High quality of care is important in PD, and limited experience in the renal unit may limit the use of PD as the initial treatment modality. According to our findings, some patients may then be deprived of a superior treatment.

In addition to the observational design of the study, some limitations need to be mentioned. Analyses were restricted to existing baseline variables in the Norwegian Renal Registry at the time of dialysis initiation. Thus, we were not able to match patients with regard to variables not included in the registry. Time in dialysis is usually short in Norway mainly due to high transplantation rates. The median time of follow-up was shorter in our study compared to what others have observed [9, 12, 15, 24]. Shorter median follow-up time could have affected our statistical power to predict late complications of any dialysis modality. Furthermore, the propensity matched analyses included only HD patients with similar baseline characteristics as patients actually treated with PD in the time period. As patients with baseline characteristics typically associated with HD are not included in our analyses, generalization and applicability of our results to apply for all ESRD patients should be done with care.

The choice of dialysis modality in individuals should not be decided solely on mortality studies [26]. Patient characteristics and preferences as well as, patient-related outcomes and centre experience may impact the choice [27, 28]. The use of PD has decreased in the western world, leaving HD the prominent initial dialysis modality in most countries [29, 30]. It is a paradox that less expensive equipotent treatment that may even provide improved prognosis in selected patients is not more widely preferred as initial ESRD treatment.

## Conclusions

In this propensity matched study, PD as initial dialysis modality was not inferior to HD concerning five-year all-cause or cardiovascular mortality in any investigated subgroup of patients in the Norwegian dialysis population. PD as initial dialysis modality conveyed favourable survival compared to HD in patients younger than 65 years. Opposed to the trend of exaggerated use of HD compared to PD, increased use of PD could be advocated according to our data.

## Abbreviations

COD: Cause of death
CVD: Cerebrovascular disease
eGFR: Estimated glomerular filtration rate
ESRD: End stage renal disease
HD: Hemodialysis
LVH: Left ventricular hypertrophy
PAD: Peripheral arterial disease
PD: Peritoneal dialysis
RRT: Renal replacement therapy
TIA: Transischemic attack

## References

[1] Annual Report 2011, the Norwegian Renal Registry [http://www.nephro.no/nnr/AARSM2011.pdf]

[2] Eggers PW (2011). Has the incidence of end-stage renal disease in the USA and other countries stabilized?. Curr Opin Nephrol Hypertens.

[3] Li PK, Chow KM (2013). Peritoneal dialysis-first policy made successful: perspectives and actions. Am J Kidney Dis.

[4] van de Luijtgaarden MWM, Jager KJ, Stel VS, Kramer A, Cusumano A, Elliott RF, Geue C, MacLeod AM, Stengel B, Covic A (2013). Global differences in dialysis modality mix: the role of patient characteristics, macroeconomics and renal service indicators. Nephrol Dial Transplant.

[5] Ortiz A, Covic A, Fliser D, Fouque D, Goldsmith D, Kanbay M, Mallamaci F, Massy ZA, Rossignol P, Vanholder R (2014). Epidemiology, contributors to, and clinical trials of mortality risk in chronic kidney failure. Lancet.

[6] Foley RN, Parfrey PS, Sarnak MJ (1998). Clinical epidemiology of cardiovascular disease in chronic renal disease. AmJ Kidney Dis.

[7] Yeates K, Zhu N, Vonesh E, Trpeski L, Blake P, Fenton S (2012). Hemodialysis and peritoneal dialysis are associated with similar outcomes for end-stage renal disease treatment in Canada. Nephrol Dial Transplant.

[8] Mehrotra R, Chiu YW, Kalantar-Zadeh K, Bargman J, Vonesh E (2011). Similar outcomes with hemodialysis and peritoneal dialysis in patients with end-stage renal disease. Arch Intern Med.

[9] Chang YK, Hsu CC, Hwang SJ, Chen PC, Huang CC, Li TC, Sung FC (2012). A comparative assessment of survival between propensity score-matched patients with peritoneal dialysis and hemodialysis in Taiwan. Medicine.

[10] Quinn RR, Hux JE, Oliver MJ, Austin PC, Tonelli M, Laupacis A (2011). Selection bias explains apparent differential mortality between dialysis modalities. J Am Soc Nephrol.

[11] Vonesh EF, Snyder JJ, Foley RN, Collins AJ (2006). Mortality studies comparing peritoneal dialysis and hemodialysis: What do they tell us?. Kidney Int.

[12] Weinhandl ED, Foley RN, Gilbertson DT, Arneson TJ, Snyder JJ, Collins AJ (2010). Propensity-matched mortality comparison of incident hemodialysis and peritoneal dialysis patients. J Am Soc Nephrol.

[13] Lukowsky LR, Mehrotra R, Kheifets L, Arah OA, Nissenson AR, Kalantar-Zadeh K (2013). Comparing mortality of peritoneal and hemodialysis patients in the first 2 years of dialysis therapy: a marginal structural model analysis. Clin J Am Soc Nephrol.

[14] Kumar VA, Sidell MA, Jones JP, Vonesh EF (2014). Survival of propensity matched incident peritoneal and hemodialysis patients in a United States health care system. Kidney Int.

[15] van de Luijtgaarden MW, Noordzij M, Stel VS, Ravani P, Jarraya F, Collart F, Schon S, Leivestad T, Puttinger H, Wanner C (2011). Effects of comorbid and demographic factors on dialysis modality choice and related patient survival in Europe. Nephrol Dial Transplant.

[16] McDonald SP, Marshall MR, Johnson DW, Polkinghorne KR (2009). Relationship between dialysis modality and mortality. J Am Soc Nephrol.

[17] Austin PC (2011). An Introduction to Propensity Score Methods for Reducing the Effects of Confounding in Observational Studies. Mult Behav Res.

[18] ERA-EDTA Registry: Annual Report 2012 [http://www.era-edta-reg.org/index.jsp?p=14]

[19] Thoemmes F: Propensity score matching in SPSS. 2012 http://arxiv.org/ftp/arxiv/papers/1201/1201.6385.pdf.

[20] Chaudhary K, Sangha H, Khanna R (2011). Peritoneal dialysis first: rationale. Clin J Am Soc Nephrol.

[21] Heaf JG, Wehberg S (2014). Relative survival of peritoneal dialysis and haemodialysis patients: effect of cohort and mode of dialysis initiation. PLoS One.

[22] Yohanna S, Alkatheeri AMA, Brimble SK, McCormick B, Iansavitchous A, Blake PG, Jain AK (2015). Effect of Neutral-pH, Low–Glucose Degradation Product Peritoneal Dialysis Solutions on Residual Renal Function, Urine Volume, and Ultrafiltration: A Systematic Review and Meta-Analysis. Clin J Am Soc Nephrol.

[23] Garcia-Lopez E, Lindholm B, Davies S (2012). An update on peritoneal dialysis solutions. Nat Rev Nephrol.

[24] Kim H, Kim KH, Park K, Kang SW, Yoo TH, Ahn SV, Ahn HS, Hann HJ, Lee S, Ryu JH (2014). A population-based approach indicates an overall higher patient mortality with peritoneal dialysis compared to hemodialysis in Korea. Kidney Int.

[25] Davies SJ (2013). Peritoneal dialysis[mdash]current status and future challenges. Nat Rev Nephrol.

[26] Dalal P, Sangha H, Chaudhary K (2011). In Peritoneal Dialysis, Is There Sufficient Evidence to Make "PD First" Therapy?. Int J Nephrol.

[27] Power A, Brown E (2013). Optimising treatment of end-stage renal disease in the elderly. Nephron Clin Pract.

[28] Da Silva-Gane M, Wellsted D, Greenshields H, Norton S, Chandna SM, Farrington K (2012). Quality of Life and Survival in Patients with Advanced Kidney Failure Managed Conservatively or by Dialysis. Clin J Am Soc Nephrol.

[29] Stengel B, Billon S, Van Dijk PC, Jager KJ, Dekker FW, Simpson K, Briggs JD (2003). Trends in the incidence of renal replacement therapy for end-stage renal disease in Europe, 1990-1999. Nephrol Dial Transplant.

[30] Mehrotra R (2007). Changing patterns of peritoneal dialysis utilization in the United States. Perit Dial Int.

